# Managing Complications Following Endoscopic Myotomy as a Treatment for Upper Esophageal Sphincter (UES) Achalasia: A Case Report

**DOI:** 10.7759/cureus.30613

**Published:** 2022-10-23

**Authors:** Pierce L Claassen, Connor J Eggleston, Nicolas A Villa

**Affiliations:** 1 Medicine, Washington State University Elson S. Floyd College of Medicine, Spokane, USA; 2 Medicine, Creighton University School of Medicine, Omaha, USA; 3 Gastroenterology, MultiCare Valley Hospital, Spokane Valley, USA

**Keywords:** esophago-gastro-duodenoscopy, esophageal dilation, esophageal stenosis, esophageal stricture, upper esophageal sphincter, endoscopy, perforation, esophagus, dysphagia, achalasia

## Abstract

Achalasia is a chronic gastrointestinal disorder characterized by increased esophageal sphincter tone and dysmotility that causes worsening dysphagia. While this condition usually affects the lower esophageal sphincter, we present a rare case of upper esophageal sphincter (UES) achalasia of unknown etiology in a female in her sixth decade of life. This was managed via UES myotomy but was complicated by esophageal perforation and severe post-operative stenosis. Consequently, the patient was referred to gastroenterology and treated over the course of two months with six endoscopic dilatations and glucocorticoid injections. Few cases of idiopathic UES achalasia have been described to date.

## Introduction

Idiopathic cricopharyngeal dysfunction, or UES achalasia, is a rare clinical entity. While there is much data available that describe the management strategies for achalasia caused by dysfunction of the lower esophageal sphincter, there is a paucity of information regarding the treatment of UES achalasia. Given this, the treatment modalities can vary widely depending on the institutional standard of care and individual physician preference. With this variation comes unpredictable procedural efficacy and an inherently increased risk of complications. Here, we present a case of UES achalasia that was successfully treated via UES myotomy by otolaryngology. Unfortunately, following the procedure, the patient developed an esophageal perforation. Although this perforation was managed conservatively, an esophageal stricture formed and eventually caused severe dysphasia, weight loss, and malnutrition. The patient was then referred to an advanced endoscopy-trained gastroenterologist who managed the esophageal stenosis with serial dilations and local steroid injections. This case report was presented at the American College of Gastroenterology Annual Meeting on October 25th, 2022 in Charlotte, North Carolina.

## Case presentation

**Figure 1:**
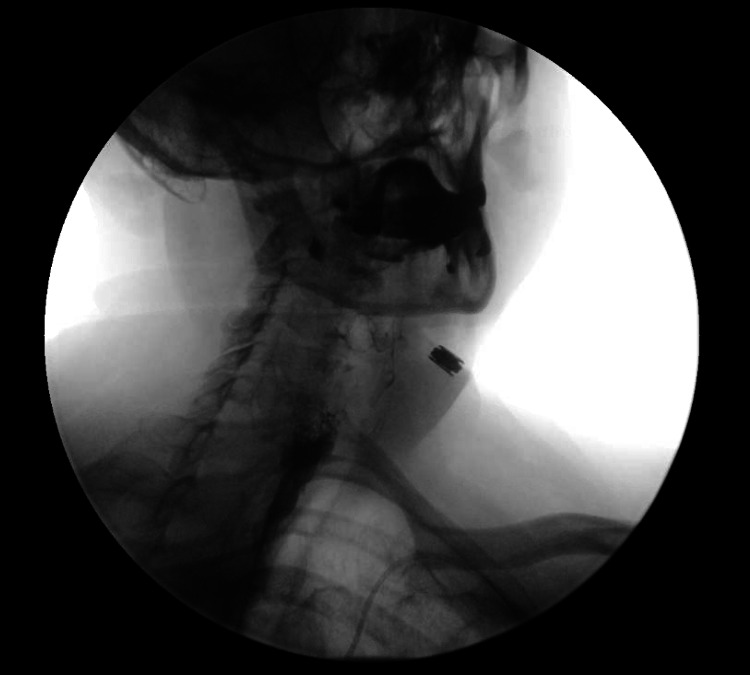
Barium swallow X-ray image taken at the end of the patient's hospital stay. Image shows no evidence of contrast leak from the esophagus, indicating that the prior esophageal perforation had healed.

A woman in her sixth decade of life presented to the gastroenterology clinic two months after undergoing a UES endoscopic myotomy by otorhinolaryngology, a common surgical treatment for idiopathic UES achalasia. While this procedure successfully treated the achalasia, it was complicated by esophageal perforation. One day post-surgery, she was admitted to the hospital with facial and neck swelling, severe pain, and difficulty swallowing. CT showed proximal esophageal perforation and subcutaneous emphysema that extended from the mediastinum to the orbits. The patient was made nothing per os (NPO) and conservatively managed with IV steroids and antibiotics. On hospital day 3, the placement of a nasogastric tube failed, so she was put on total parenteral nutrition (TPN). By hospital day 7, her edema, crepitus, pain, and dysphagia improved. Barium swallow showed no contrast leak from the esophagus, so TPN was discontinued, and she was discharged (Figure 1).

**Figure 2:**
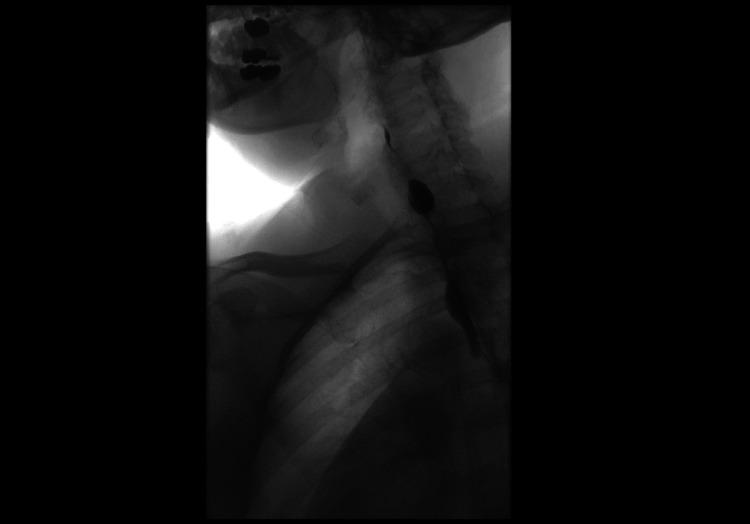
Barium swallow X-ray image taken one month following the patient's hospital stay. Image shows evidence of contrast pooling proximally to a focal esophageal stricture.

Following this, the patient developed progressive dysphagia so additional imagining was ordered to investigate probable esophageal stenosis. Barium swallow showed a severe, short segment stricture at the proximal esophagus that did not change with swallowing, so she was referred to advanced endoscopy for further evaluation (Figure 2). Upon arrival at the gastroenterology clinic, she reported severe dysphagia to both solids and liquids, noting a 50-pound weight loss since the UES myotomy. It was determined by the care team that urgent endoscopic evaluation was warranted. 

**Figure 3:**
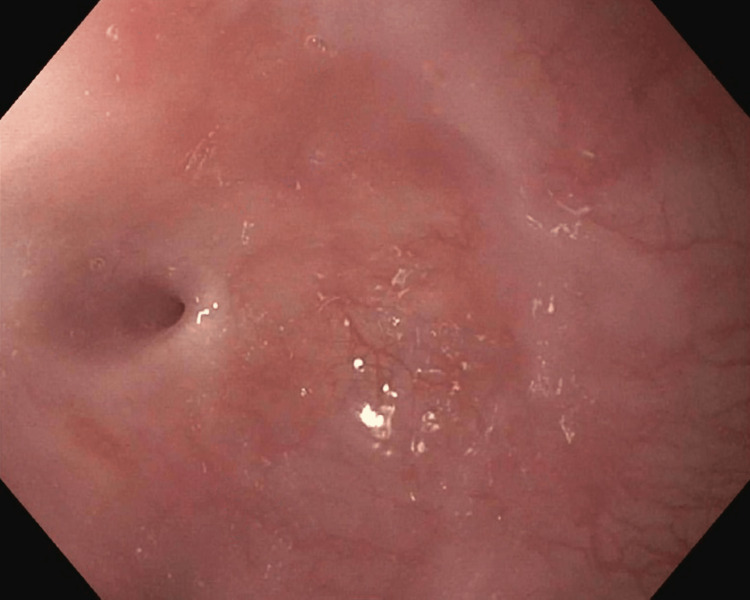
Anterograde view of the esophageal lumen proximal to the upper esophageal sphincter, 15 cm distal to the upper incisors. Circumferential, full-thickness constriction reduced the luminal diameter to 2 mm at the narrowest point. The image was taken with an esophagogastroduodenoscope during the patient’s first visit to the advanced endoscopy gastroenterology clinic.

During the first esophagogastroduodenoscopy (EGD), the esophagus was found to be focally stenotic (15 cm distal to the upper incisors at the level of the UES) with the lumen being reduced to 2 mm in diameter at its narrowest point (Figure 3). Due to the morbidity of the stenosis, the decision was made to immediately increase the luminal size via endoscopic dilation. 

**Figure 4:**
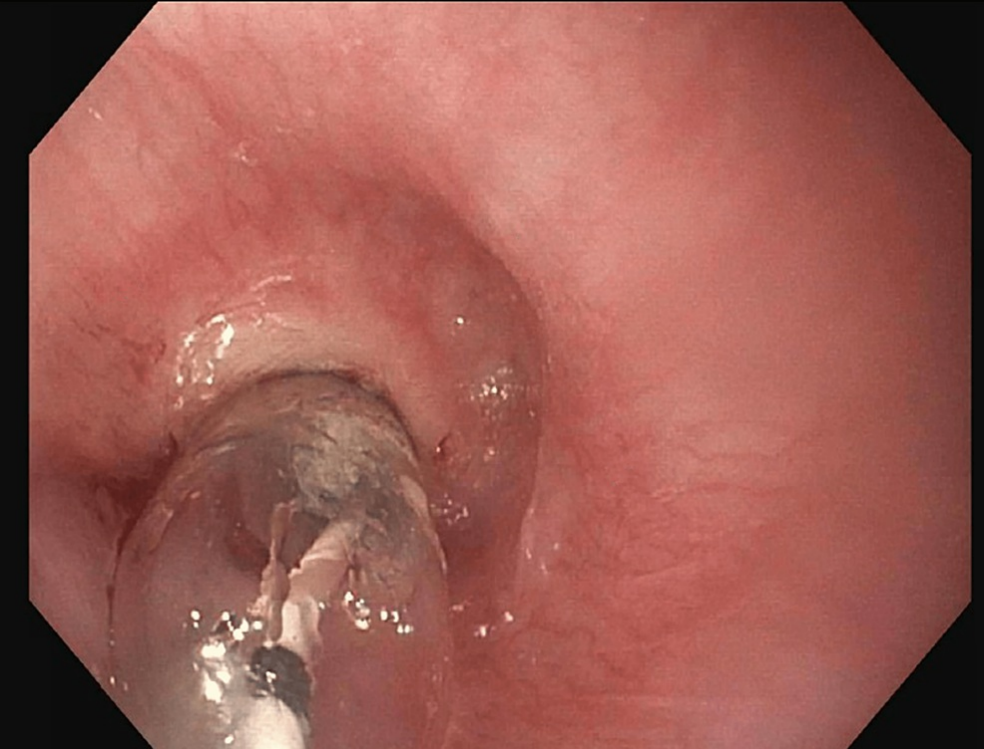
Anterograde view of the esophageal lumen proximal to the upper esophageal sphincter, 15 cm distal to the upper incisors. A 6 mm TTS balloon biliary dilator was inflated, dilating the esophageal stricture. The image was taken with an esophagogastroduodenoscope during the patient’s first visit to the advanced endoscopy gastroenterology clinic. TTS, through-the-scope

**Figure 5:**
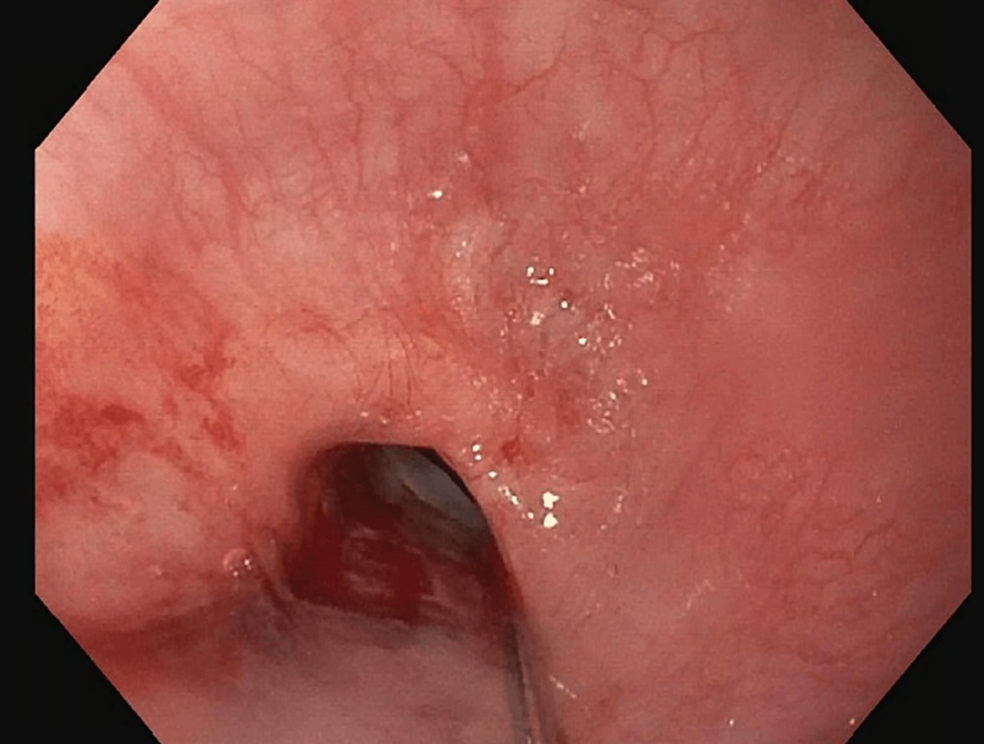
Anterograde view of the esophageal lumen proximal to the upper esophageal sphincter, 15 cm distal to the upper incisors, after successfully passing a 24 French (8 mm) Savary dilator. The image was taken with an esophagogastroduodenoscope during the patient’s first visit to the advanced endoscopy gastroenterology clinic.

After a failed first attempt to dilate with a 15 French Savary dilator, an ultra-small, through-the-scope (TTS) balloon dilator was retrieved. Under fluoroscopic guidance, a guidewire was passed through the UES, and a 6-mm TTS balloon biliary dilator was successfully inflated (Figure 4). Larger TTS balloons were progressively introduced, then a 24 French Savary dilator was passed, safely enlarging the lumen to 8 mm in diameter at the conclusion of the first EGD (Figure 5). Due to the patient’s poor nutrition status and the necessity for additional dilations, she was referred to interventional radiology for urgent percutaneous endogastric (PEG) tube placement.

**Figure 6:**
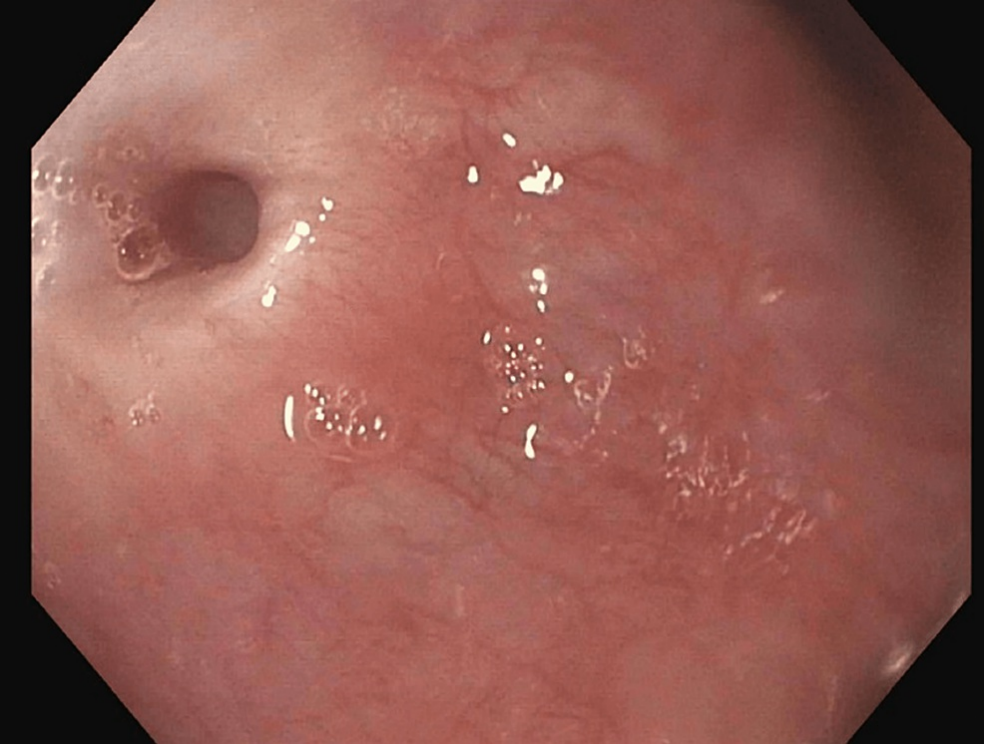
Anterograde view of the esophageal lumen proximal to the upper esophageal sphincter. Circumferential constriction at 15 cm distal to the upper incisors reduced the luminal diameter to 3 mm at the narrowest point. The image was taken with an esophagogastroduodenoscope during the patient’s second visit to the advanced endoscopy gastroenterology clinic.

**Figure 7:**
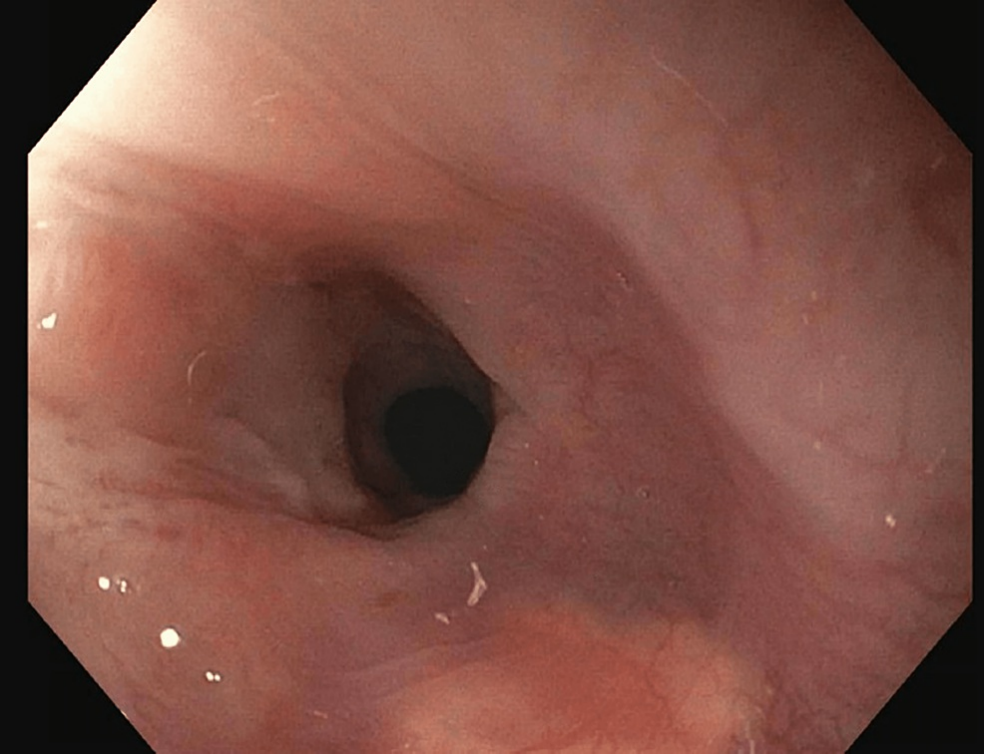
Anterograde view of the esophageal lumen proximal to the upper esophageal sphincter. Circumferential constriction at 15 cm distal to the upper incisors reduced the luminal diameter to 6 mm at the narrowest point. The image was taken with an esophagogastroduodenoscope during the patient’s third visit to the advanced endoscopy gastroenterology clinic.

**Figure 8:**
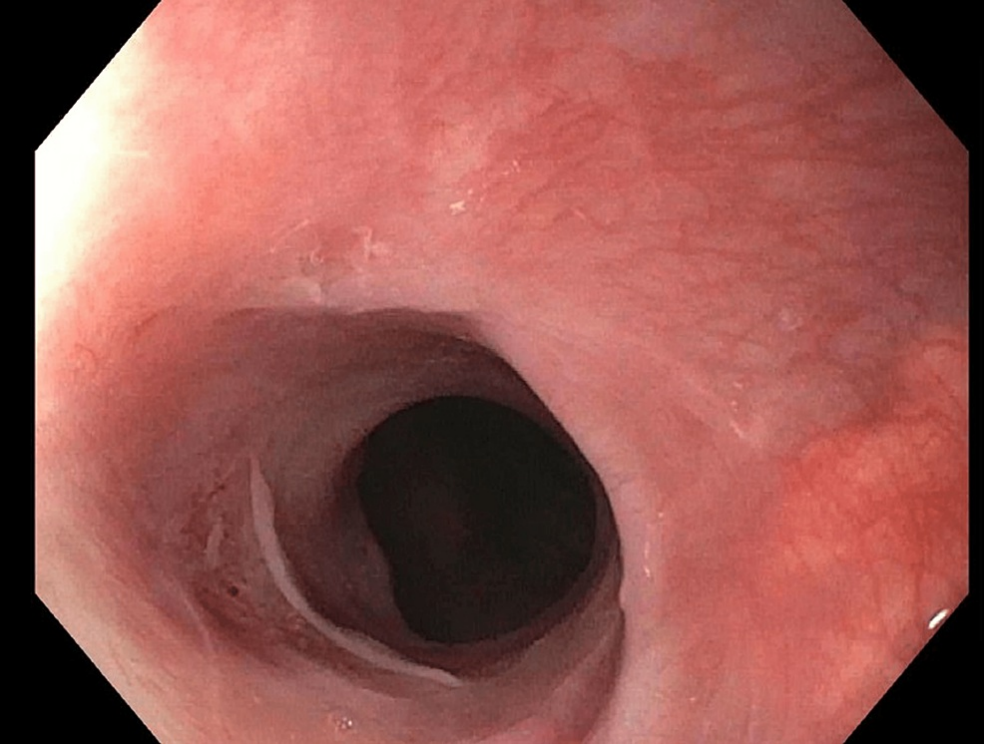
Anterograde view of the esophageal lumen proximal to the upper esophageal sphincter. Circumferential constriction at 15 cm distal to the upper incisors moderately reduced the luminal diameter. The image was taken with an esophagogastroduodenoscope during the patient’s fourth visit to the advanced endoscopy gastroenterology clinic.

**Figure 9:**
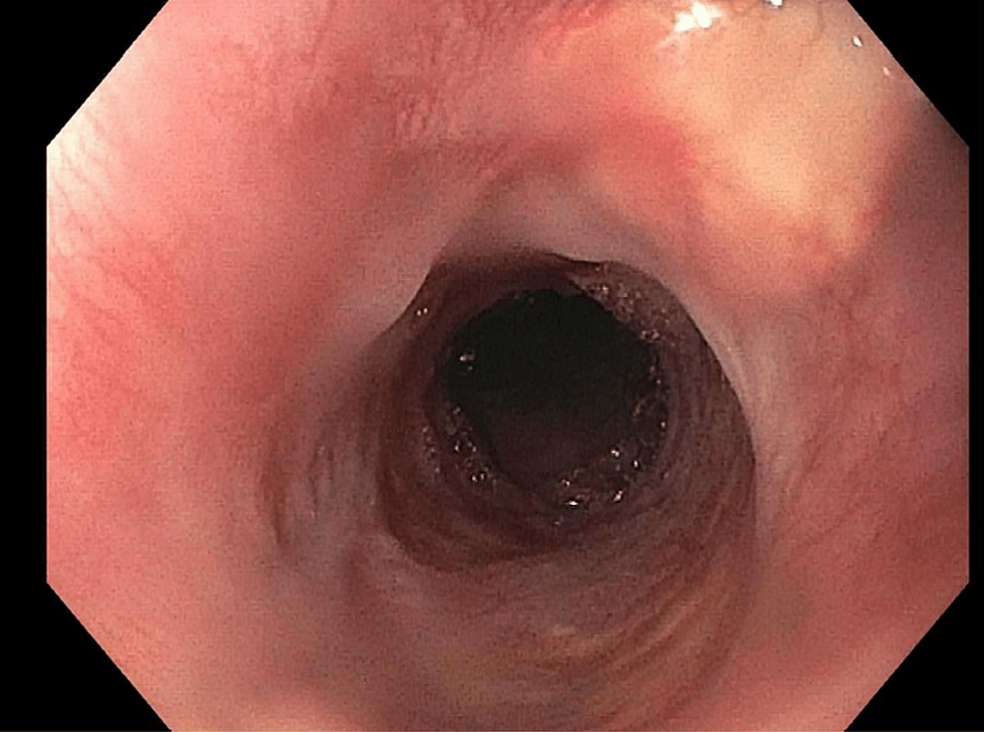
Anterograde view of the esophageal lumen proximal to the upper esophageal sphincter. Circumferential constriction at 15 cm distal to the upper incisors moderately reduced the luminal diameter. The image was taken with an esophagogastroduodenoscope during the patient’s fifth visit to the advanced endoscopy gastroenterology clinic.

**Figure 10:**
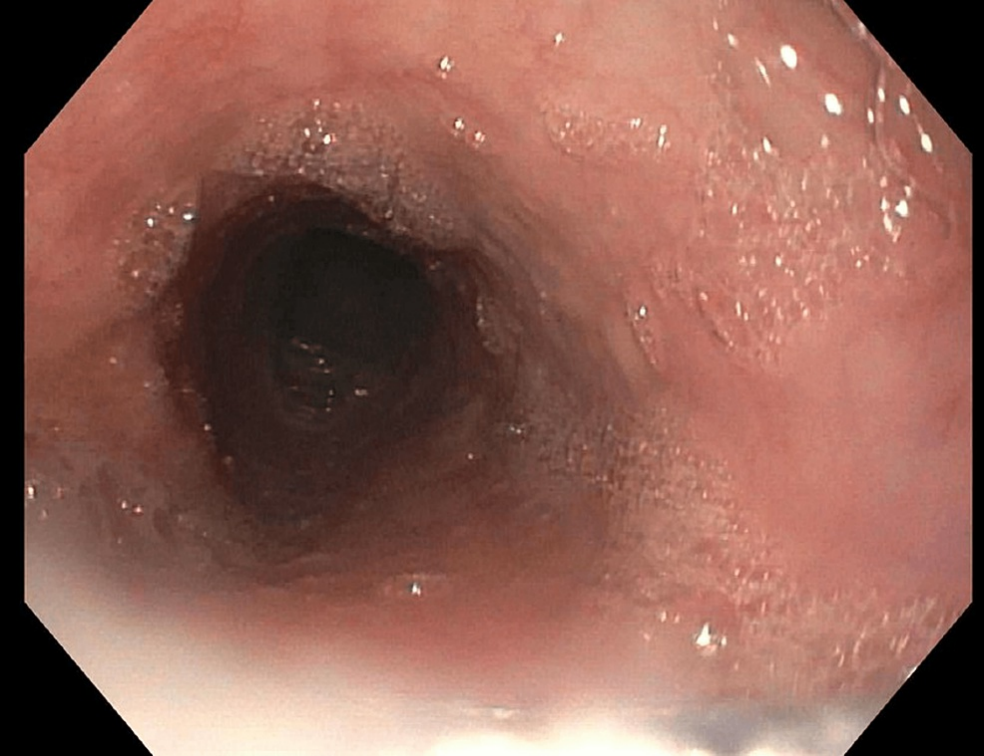
Anterograde view of the esophageal lumen proximal to the upper esophageal sphincter. Circumferential constriction at 15 cm distal to the upper incisors mildly reduced the luminal diameter. The image was taken with an esophagogastroduodenoscope during the patient’s sixth and final visit to the advanced endoscopy gastroenterology clinic.

During five subsequent EGDs, TTS balloon and Savary dilators were serially increased in size to ultimately expand the luminal diameter to 20 mm, 60 French (Figures 6-10). Local triamcinolone injections were also circumferentially administered (2 mL at 40 mg/mL) to minimize the risk of fibrosis and restenosis. While in the gastroenterology clinic for her sixth and final EGD, the patient reported tolerating regular food and regaining weight, so her PEG tube was removed. This sixth and final dilation occurred two months after her initial presentation. She was instructed to resume her usual diet and contact the gastroenterology clinic if her symptoms returned. 

## Discussion

This rare case of idiopathic cricopharyngeal achalasia represents an uncommon cause of severe dysphagia in an otherwise healthy adult without a history of stroke, radiation therapy, or a congenital predisposition for pharyngoesophageal dysfunction. While endoscopic esophageal myotomy is a recommended definitive treatment for this condition, the possibility of procedure-induced complications exists and must not be overlooked [1-2]. Managing postoperative esophageal stenosis will likely be the responsibility of the gastroenterology care team. Although this complication occurs infrequently, it can be severe in nature, so rapid stricture identification is imperative. Early management of post-myotomy esophageal stenosis should be done endoscopically to identify the degree of the stricture and perform a controlled initial dilation under fluoroscopic guidance [3]. Subsequent serial dilations can then be performed with balloon dilators, Savary dilators, or a combination of both. Studies have shown that administering local corticosteroid injections can help prevent post-dilation fibrosis and subsequent esophageal restenosis [4]. 

If obstructive strictures are to recur, counseling patients about the possibility of self-administered esophageal dilation may be a practical consideration. There is evidence supporting the efficacy and safety of self-dilation in patients with benign, refractory esophageal strictures [5]. This technique may help to preserve esophageal function, thereby prolonging a patient’s need to undergo future endoscopic dilatation. This style of management will likely reduce healthcare utilization, which may be particularly valuable in rural and underserved areas where patients often travel long distances to receive specialty healthcare services from providers who work in congested medial systems.

## Conclusions

Complications such as stricture formation following cricopharyngeal myotomy must be identified and treated urgently. The resulting esophageal stenosis can be morbid in nature and greatly increase a patient’s risk of aspiration, immense weight loss, and associated malnutrition. For the therapeutic endoscopist, there are effective treatment options available to rapidly improve these patient’s nutrition status and quality of life.
